# Supplementary material for: Decoding the Hot‐Mitochondrion Paradox
Source: Bioessays. 2026 Jul 9;48(7):e70159. doi: 10.1002/bies.70159 (PMC13347768; doi:10.1002/bies.70159)
Supplement: Supplementary file 1 — Supporting File 1: bies70159‐sup‐0001‐SuppMat.docx. [file BIES-48-e70159-s002.docx]

**Supporting Information**

**Impact of parameter variations on ATP-synthase-driven temperature spikes:**

To estimate the magnitude of ATP-synthase temperature spikes using Eq. (1), we initially used fixed parameter values in the main text, which yield a single temperature-difference solution. However, because these parameters vary across the literature depending on physiological conditions and experimental techniques, we now provide the full reported ranges and subsequently examine how variations in these parameters influence the temperature predicted by Eq. (1). The proton-translocation rate through ATP synthase, $N_{p.u.t.}$, spans approximately 800 – 3500 s^-1^ [1,2]. The effective torsional stiffness of the γ-subunit ranges from 82 to 364 pN.nm.rad^-2^ [3]. The combined Gibbs free energy of protonation and hydration of protons has been reported between 1024 and 1284 kJ.mol^-1^ [4]. Finally, the rotational friction coefficient, expressed as $\lambda=k_{B}T/D$, corresponds to diffusion coefficients reported in MD simulations of $D$ = 0.001 – 0.003 rad^2^.ns^-1^ [5]. We selected the parameter values used in the main text as the mean values, and then performed Monte Carlo sampling (25,000 iterations) assuming Gaussian variability around each mean. The resulting distribution is shown in the Supporting Information Figure 1, yielding a minimum, maximum, and mean temperature difference spike of approximately 2 °C, 37 °C, and 6.5 °C, respectively. Panels B–E show how each parameter correlates with the resulting temperature spike across the full Monte Carlo sample, where all parameters vary simultaneously. Thus, each panel illustrates the overall relationship between a single parameter and Δ*T* while the remaining parameters fluctuate within their uncertainty ranges.


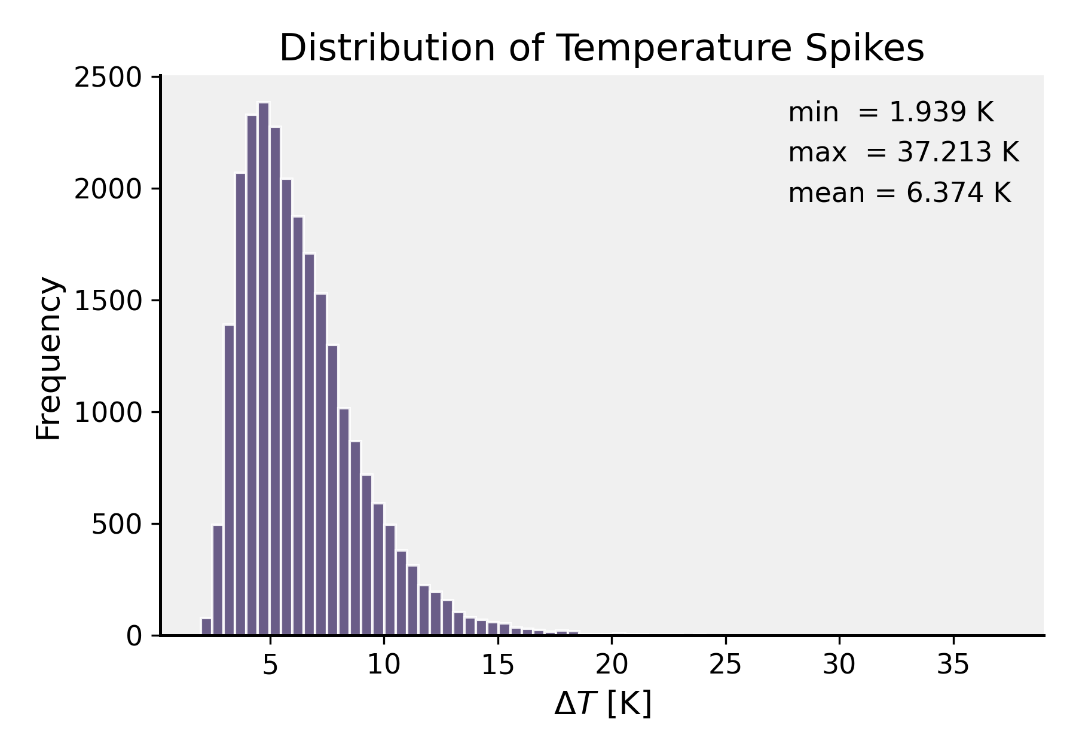


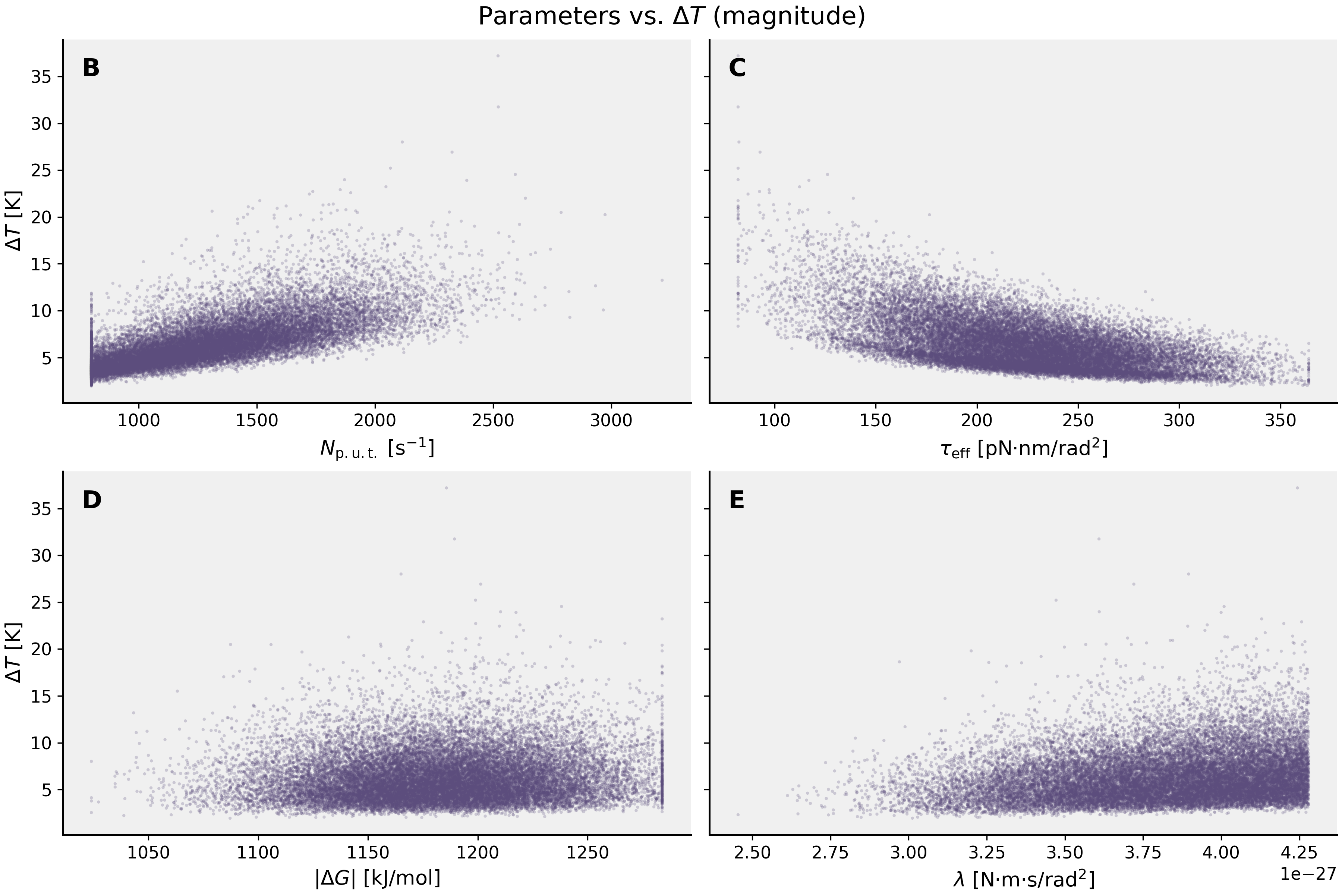


Supporting Information Fig. 1: Resolution of the paradox: using the values in the main text as central estimates, 25,000 Monte Carlo draws (Gaussian distribution) produce temperature differences of ~2–37 °C, with an average of ~6.5 °C. Panels B–E illustrate how modifying each parameter on its own alters the predicted temperature rise.

**Population of active ATP synthases per mitochondrion:**

The metabolic rate of an active aerobic amoeba (*Saccamoeba limax*) with a body mass of 5.15×10^-9^ g has been reported as 2.89×10^-9^ W [6]. Assuming a mass density equal to that of water, this corresponds to a cell volume of 5.15×10^3^ μm^3^. According to Fahimi *et al.*’s scaling relationship for mitochondrial abundance in heterotrophic unicellular eukaryotes [7,8], $\log_{10} N_{\mathrm{mito}}=1.02\left( \log_{10} V-0.53 \right)$, where $V$ is the cell volume in μm^3^. For $V$ = 5.15×10^3^ μm^3^, the estimated mitochondrial population is therefore $N_{\mathrm{mito}}\approx$ 1.76×10^3^. If 90% of the organism’s metabolic rate is generated via mitochondrial oxidative phosphorylation, and 20% of that flux is dissipated as proton leak, then the remaining power available for ATP synthesis per mitochondrion is: [0.9×0.8×(2.89×10^-9^ W) / 1760] = 1.18×10^-12^ W. The population of ATP synthase complexes can then be estimated using [9]: $N_{ATP synthase}=$ (1.18×10^-12^ W) / $\left[ N_{p.u.t.}F\left( \mathrm{PMF} \right) \right]$ where $N_{p.u.t.}=1200 H^{+}/s$, $F=$ 1.6×10^-19^ J/proton/volt, and $\mathrm{PMF}$ = 0.2 V. This yields an estimated ATP synthase population of approximately 3.1×10^4^ complexes per mitochondrion.

**Tables of Abbreviations and Symbols:**

| **Abbreviation** | **Definition** |
| --- | --- |
| ANT(s) | Adenine nucleotide translocase(s) |
| ATP | Adenosine 5’-triphosphate |
| GFP | Green fluorescent protein |
| HMP | Hot-mitochondrion paradox |
| Hsp(s) | Heat shock protein(s) |
| IMM | Inner mitochondrial membrane |
| IMS | Intermembrane space |
| MCP(s) | Mitochondrial carrier protein(s) |
| MCU | Mitochondrial calcium uniporter |
| MD | Molecular dynamics (simulations) |
| MR | (Standard) metabolic rate |
| MTY | Mito-thermo-yellow |
| p.u.t. | Per unit time |
| PMF | Proton motive force |
| ROS | Reactive oxygen species |
| TD-DFT | Time-dependent density functional theory (DFT) |
| UCP(s) | Uncoupling protein(s) |
| WWs | Water wires |

| **Symbol** | **Definition** |
| --- | --- |
| Δ*G* | Gibbs free energy change associated with ion translocation |
| Δ*H* | Enthalpy change associated with ion translocation |
| Δ*S* | Entropy change associated with ion translocation |
| Δ*T* | Local transient temperature change |
| $\kappa$ | Thermal conductivity coefficient |
| $\dot{Q}$ | Rate of heat production (SI units: watts). |
| *L* | Characteristic length |
| *k_B_* | Boltzmann’ constant |
| $\tau_{eff.}$ | The effective torsional stiffness of the axel of ATP synthase |
| $\lambda$ | Coefficient of friction between the enzyme subunits and their local environments during conformational changes |
| *D* | Diffusion coefficient of ɣ-subunit |
| *N*_p.u.t._ | Number of protons translocated per unit time |
| **P** | Total ion flux per mitochondrion |
| *P_i_* | Total ion flux per Protein type *i* |
| $\dot{N}_{i}$ | Ion translocation per second per single protein *P_i_* |
| *M_i_* | Copy number of protein *P_i_* |
| *n* | Total count of protein types contributing to the observed signal |
| $\Theta$ | Cumulative thermal occupancy |
| $\tau_{i}$ | Lifetime of temperature spike |

**References**

[1] J.E. Walker, The ATP synthase: the understood, the uncertain and the unknown, *Biochem Soc Trans* **41** (2013) 1–16.

[2] H. Ueno, T. Suzuki, K. Kinosita Jr, M. Yoshida, ATP-driven stepwise rotation of F_o_F_1_-ATP synthase, *Proc Natl Acad Sci USA* **102** (2005) 1333–1338.

[3] D. Okuno, R. Iino, H. Noji, Stiffness of γ subunit of F_1_-ATPase, *Eur Biophys J* **39** (2010) 1589–1596.

[4] A. Malloum, J.J. Fifen, J. Conradie, Determination of the absolute solvation free energy and enthalpy of the proton in solutions, *J Mol Liq* **322** (2021) 114919.

[5] K.I. Okazaki, G. Hummer, Elasticity, friction, and pathway of γ-subunit rotation in F_o_F_1_-ATP synthase, *Proc Natl Acad Sci USA* **112** (2015) 10720–10725.

[6] J.P. DeLong, J.G. Okie, M.E. Moses, R.M. Sibly, J.H. Brown, Shifts in metabolic scaling, production, and efficiency across major evolutionary transitions of life, *Proc Natl Acad Sci USA* **107** (2010) 12941–12945.

[7] P. Fahimi, C.F. Matta, J.G. Okie, Are size and mitochondrial power of cells inter-determined?, *J Theor Biol* **572** (2023) 111565.

[8] J.G. Okie, V.H. Smith, M. Martin-Cereceda, Major evolutionary transitions of life, metabolic scaling and the number and size of mitochondria and chloroplasts, *Proc Roy Soc B* **283** (2016) 20160611.

[9] P. Fahimi, C.F. Matta, On the power per mitochondrion and the number of associated active ATP synthases, *Phys Biol* **18** (2021) 04LT01.
