# Supplementary figures and images for: Decoding the Hot‐Mitochondrion Paradox
Source: Bioessays. 2026 Jul 9;48(7):e70159. doi: 10.1002/bies.70159 (PMC13347768; doi:10.1002/bies.70159)

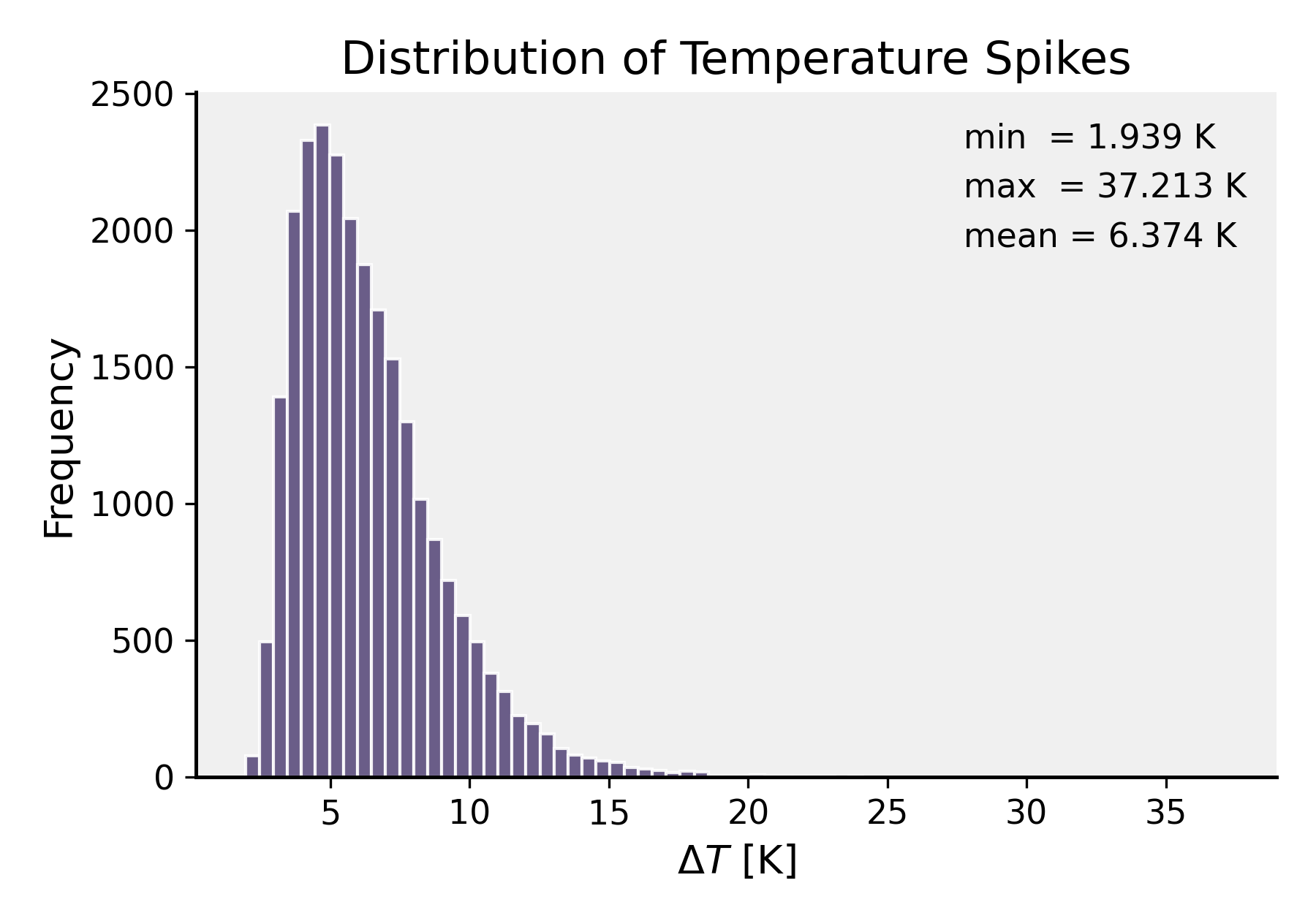

Supplement: Supplementary file 2 — Supporting File 2: bies70159‐sup‐0002‐FigureS1.zip. [file BIES-48-e70159-s001.zip › SI - Figure 1A.png]

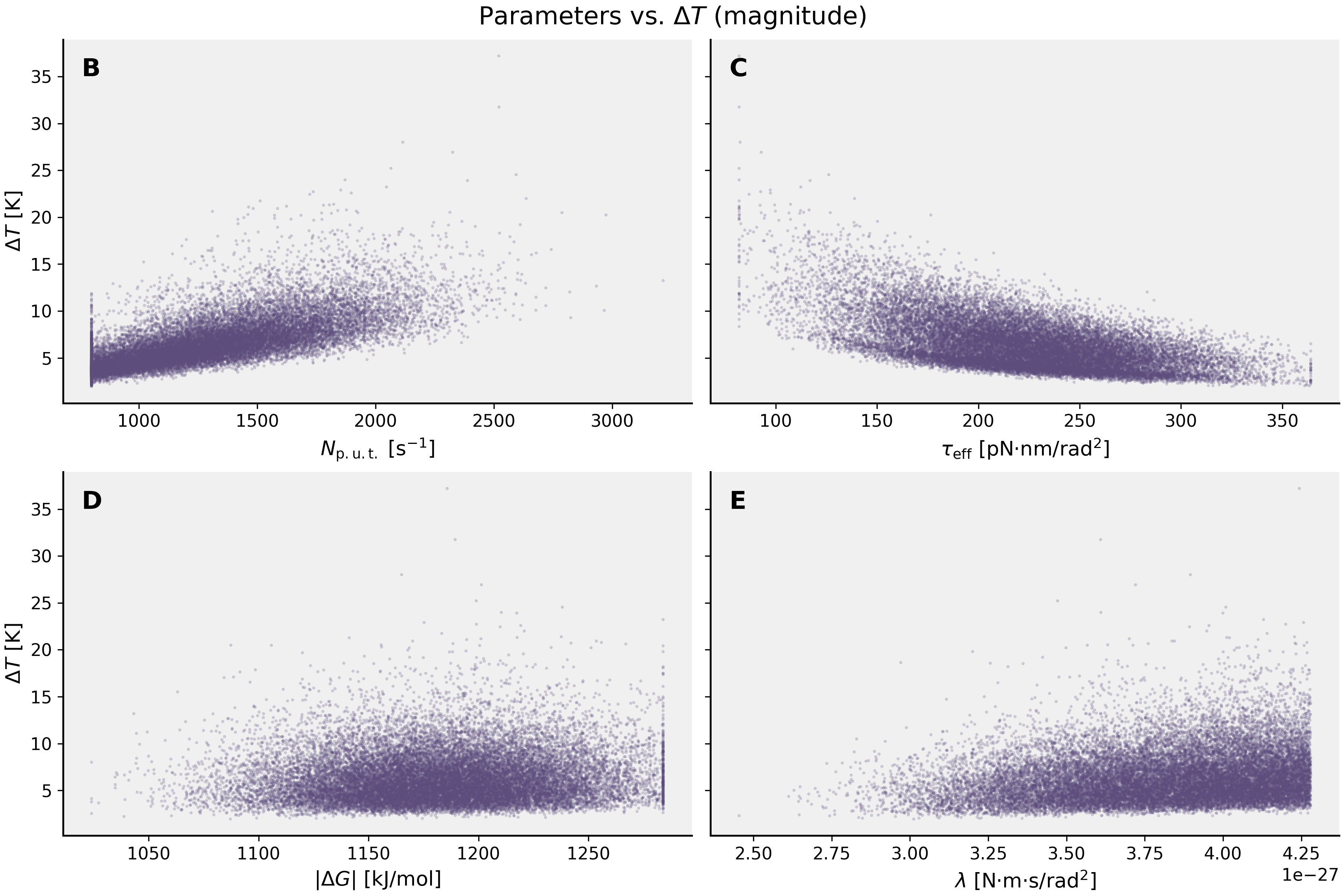

Supplement: Supplementary file 2 — Supporting File 2: bies70159‐sup‐0002‐FigureS1.zip. [file BIES-48-e70159-s001.zip › SI - Figure 1B-E.png]
